# Effect of Citric Acid on Prolonging the Half-life of Dissolved Ozone in Water

**DOI:** 10.14252/foodsafetyfscj.D-19-00005

**Published:** 2019-12-27

**Authors:** Yoshichika Hirahara, Kazuyoshi Iwata, Katsuhiko Nakamuro

**Affiliations:** 1 Kinki Regional Bureau of Health and Welfare, Oe Bldg., 7F, 1-1-22, Noninbashi, Chuo ward, Osaka 540-0011, Japan; 2 Setsunan University, 17-8, Ikedanakamachi, Neyagawa, Osaka 572-8508, Japan

**Keywords:** ozonized water, half-life, citric acid, hydroxyl radical, hydrogen abstraction

## Abstract

To elucidate the effect of citric acid on the stability of dissolved ozone, half-lives of ozone in a citric acid solution was investigated. Prolongation of the half-life of ozone was clearly shown in the presence of citric acid in ozonized water. In the presence of ethylenediaminetetraacetic acid (EDTA), the half-life of ozone was decreased. The addition of various concentrations of citric acid to the EDTA solution, however, reversed the half-life in a concentration-dependent manner. These indicate that citric acid suppresses ozone self-decomposition in water. A citric acid-mediated suppression mechanism of ozone self-decomposition involving hydroxy radical (HO•) was proposed as follows: HO• formed by the radical chain reaction process of ozone is scavenged by a way of abstracting the hydrogen atom bound to a carbon atom located α-position of a carbonyl group. The radical chain reaction of ozone is, thus, suppressed. These findings demonstrate that the addition of citric acid to ozonized water is useful for the stabilization of ozone. This ability may contribute to the application of ozone sterilization in food production processes.

## 1. Introduction

Ozone is one of the most powerful disinfectant available and is capable of causing the oxidative decomposition of many organic pollutants^1^^)^. Dissolved ozone (hereinafter referred to as ozone) is widely used in water treatment for disinfection and oxidation^2^^)^. In food processing, excess ozone automatically decomposes rapidly to produce oxygen and thus does not leave any residue in foods^3^^)^. Thus, utilization of ozone gas and ozonized water as food additives has been expanding in the food industry and is widely used for preserving fresh fish^4^^)^, corn and soybeans^5^^)^, poultry^6^^)^, smoked squid^7^^)^, and baked chikuwa^8^^)^. Ozone is also known as a food additive for suppressing the growth of yeasts and lactic acid bacteria instantly in many food manufacturing processes^9^^)^. However, ozone decays rapidly in water^10^^)^. Therefore, ozone has to be generated onsite for use in ozonized water. Thus, controlling the stability of dissolved ozone in water is important to expand the application of ozone in the food industry.

In general, ozone has high reactivity with organic substances having C═C double bonds, -SH groups, or -NH_2_ groups, but its reactivity with aliphatic saturated carboxylic acids such as acetic acid, butyric acid, oxalic acid, and citric acid is extremely low^11^^)^. Among these, citric acid is a food additive and used safely in food manufacturing processes. Many studies on the stability of citric acid are related to the decomposition of citric acid by ozone. However, little is known about the stability of ozone itself when it coexists with citric acid in water. Therefore, in this study, the stability of ozone itself in the presence of citric acid and the effect of citric acid on the half-life of dissolved ozone in water was investigated for the effective ozonized water.

## 2. Materials and Methods

### 2.1 Equipment and Reagents

Distilled water was produced using pure water manufacturing equipment (RFD240NC ADVANTEC, Toyo Roshi Kaisha, Ltd. Tokyo, Japan). Ozonized water was generated from distilled water using an ozonized water generator (Model E-5, Suiseikogyo, Inc. Ltd. Amagasaki, Japan) with a direct electrolysis method. Ozone concentration was measured using an ozone monitor (Model EL550, Ebara Jitsugyo Co., LTD. Tokyo, Japan). A pH of solution was measured by a digital ion concentration meter (Model IM-20E, TOA Electronics Ltd. Nagoya, Japan). Special grade of citric acid and ethylenediaminetetraacetic acid(EDTA)-2Na were purchased from Wako Pure Chemical Industry Ltd. Japan.

### 2.2 Preparation of Reagent Solution

Ozonized water was prepared from distilled water. Approximately 0.14 mmol/L ozonized water was added to 300 μL of 1 mol/L solution of citric acid to final volume of 300 mL. This solution was pH 3.2. Thirty μL of 100 mmol/L EDTA-2Na and each of 1500, 300, 150, 75, and 0 μL of 1 mol/L solution of citric acid were added to a 500 mL beaker, and then approximately 0.1 mmol/L ozonized water was each added to give a final volume of 300 mL. These solutions showed ozone concentrations of approximately 0.1 mmol/L, an EDTA-2Na concentration of 10 μmol/L, and each citric acid concentration of 5, 1, 0.5, 0.25, and 0 mmol/L.

Immediately after preparation, these solutions were used for ozone half-life measurements, with experiments conducted at around 20°C. After measuring the ozone concentrations with an ozone monitor, the pHs of the solutions were measured. It was confirmed that there was almost no change in pH as compared with the pH at the time of preparation.

### 2.3 Measurement Method of Dissolved Ozone-half Life

The ozone concentration in 300 mL of each sample solution was measured using absorbance at a wavelength of 253.7 nm by circulation using a silicon tube at a flow rate of 100 mL/min through the ozone monitor. The ozone concentration was measured every minute, and the half-life of ozone was obtained from these values. After the measurement, the flow cell was washed using circulating distilled water.

## 3. Results and Discussion

**Fig. 1:**
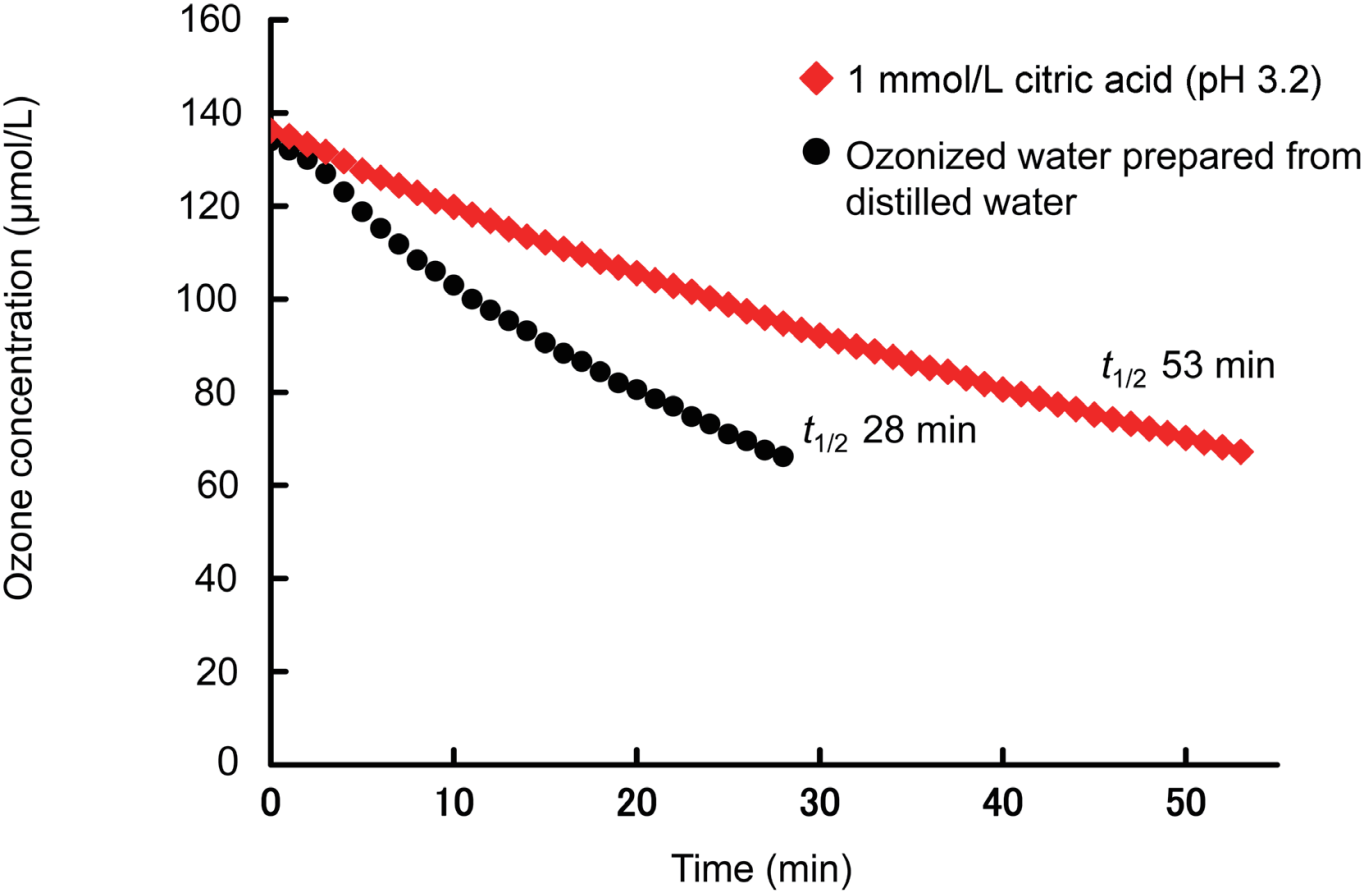
Influence of citric acid in ozonized water on the stability of ozone

**Fig. 2:**
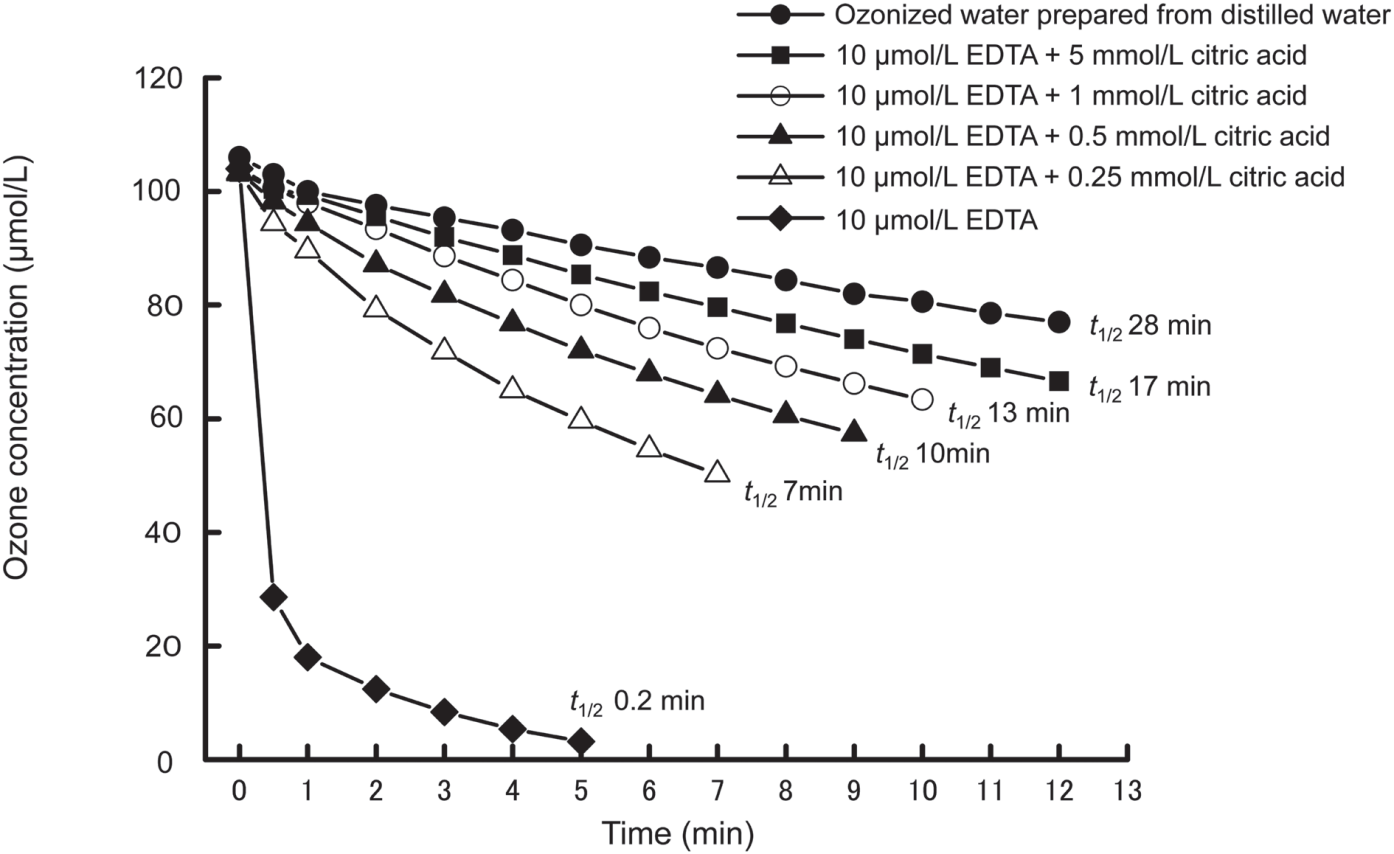
Effect of various concentrations of citric acid with 10 μmol/L EDTA on the half-life of ozone

The effect of citric acid on the stability of ozone was investigated in distilled water. The effect of citric acid on the half-life of ozone was shown in **Fig. 1**. In ozonized water prepared from distilled water, the half-life of ozone was 28 min. The half life was prolonged by about two times (53 min) in the presence of citric acid in distilled water at a concentration of 1 mmol/L, showing that citric acid contributed to the stability of ozone. Ozone is consumed readily by EDTA. The decomposition of ozone is accelerated by EDTA in aqueous solution^12^^)^. When EDTA was mixed with the ozonized water to a final concentration of 10 μmol/L, the half-life of ozone was decreased from 28 min to 0.2 min (**Fig. 2**). However, when various concentrations of citric acid were mixed with the EDTA solution, the half-life of ozone was restored with increasing citric acid concentration (**Fig. 2**), indicating that citric acid suppressed the decomposition of ozone.

**Fig. 3:**
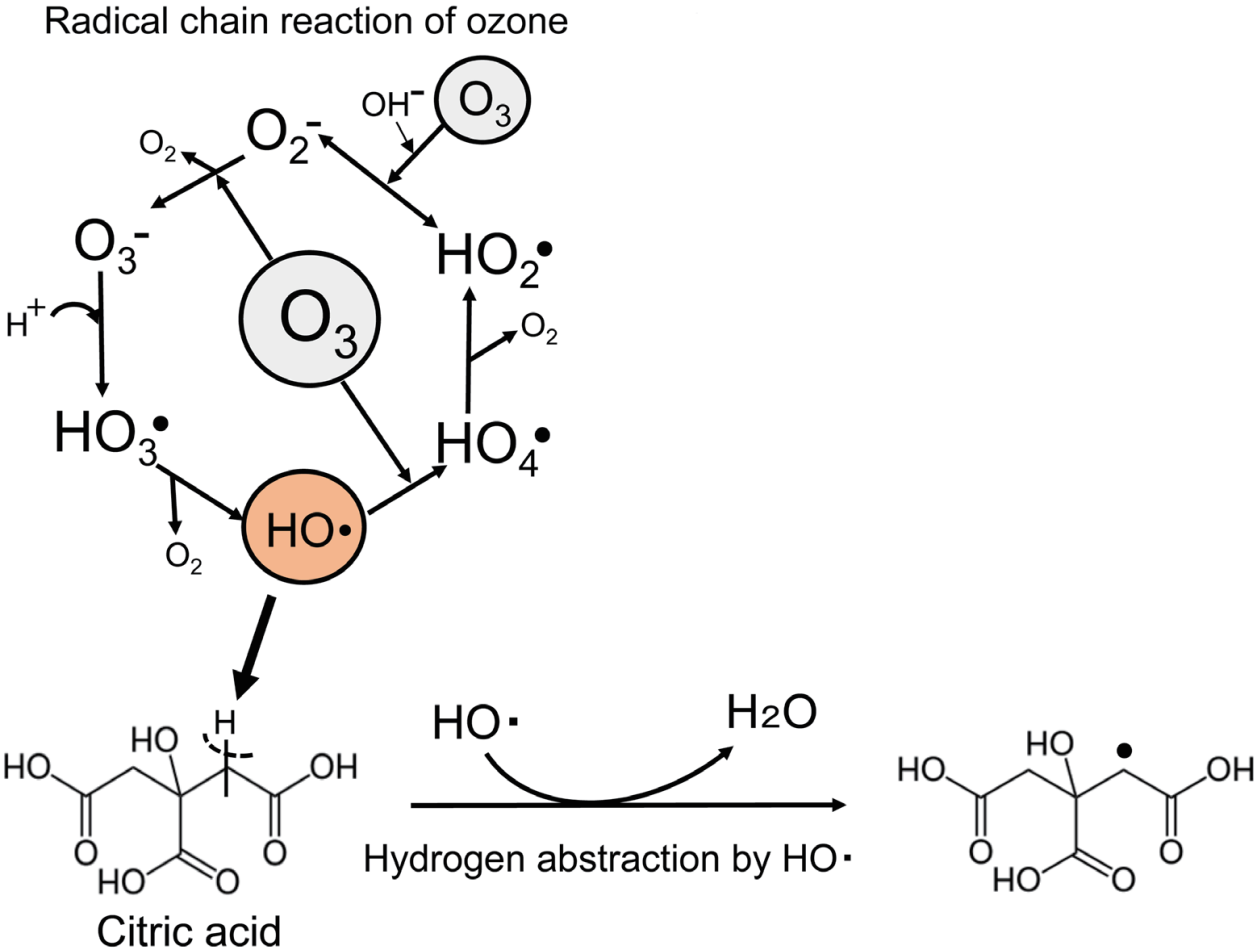
Proposed mechanism of suppression of ozone degradation with a HO• scavenging effect by citric acid involving hydrogen abstraction in a radical chain reaction

The citric acid-mediated suppressing mechanism for the decomposition of ozone is considered as follows. It is well known that the self-decomposition of aqueous ozone produces hydroxyl radicals (HO•) with a radical chain reaction with ozone^13^^–^^15^^)^. The initial step is oxygen-atom transfer from ozone to a hydroxide ion (OH-), and a hydroperoxyl radical (HO_2_•) and a superoxide anion (O_2_-) are formed. The O_2_- reacts with ozone to yield an ozonide anion (O_3_-). In acidic conditions, O_3_- combines with H^+^ to form HO_3_•, and then oxygen (O_2_) is liberated to form HO•. Therefore, HO• formation plays an important role in ozone self-decomposition in radical chain reactions. Acetic acid is known as an inhibitor of the ozone decomposition^16^^,^^17^^)^. This inhibition is due to the scavenging of HO• in the ozone self-decomposition process through abstracting a hydrogen atom bound to a carbon atom located next to a carbonyl group in acetic acid^18^^,^^19^^)^. Citric acid has three carboxyl groups, and hydrogen atoms bound to the α-position carbon atoms of the two carboxyl groups. Therefore, the HO• that forms from the self-decomposition of ozone in the radical chain reaction is presumably consumed by hydrogen abstraction from the α-carbon atoms next to the carbonyl group in citric acid, to quench the chain reaction (**Fig. 3**). Ozone self-degradation is suppressed in the radical chain reaction. Thus, the half-life of ozone would be prolonged. This hypothesis was also supported by the fact that the half-life of ozone in the presence of EDTA was prolonged by the addition of citric acid. EDTA is known to promote HO• formation in the radical chain reaction of ozone^12^^)^. In the presence of EDTA, the decomposition of ozone was accelerated. The ozone decay was, however, suppressed by the addition of citric acid, which is thought to have an inhibition effect on HO•.

In conclusion, prolongation of the half-life of ozone was observed in the presence of citric acid. The result indicates that citric acid plays an important role for the stability of ozone. Therefore, the coexistence of citric acid in ozonized water is useful for the utilization of ozone in food manufacturing processes for disinfection of foods, and to contribute to increasing the application of ozone in the food industry.
